# Challenges in transferring GP to heterogeneous contexts: the JADECARE Joint Action

**DOI:** 10.1093/eurpub/ckac129.507

**Published:** 2022-10-25

**Authors:** J Txarramendieta

**Affiliations:** Kronikgune Institute for Health Services Rese, Barakaldo, Spain

## Abstract

In this first presentation of the workshop, Jon Txarramendieta, the Project Manager at the Coordination team of JADECARE will present the Joint Action and the challenges in transferring the good practices to the heterogeneous context of the Next Adopters. It will give context to the workshop and introduce good practices that are transferred into action.

